# Dominant-negative inhibition of canonical Notch signaling in trophoblast cells does not disrupt placenta formation

**DOI:** 10.1242/bio.037721

**Published:** 2019-04-10

**Authors:** Carrie J. Shawber, Dex-Ann Brown-Grant, Tracy Wu, Jan K. Kitajewski, Nataki C. Douglas

**Affiliations:** 1 Department of Obstetrics and Gynecology, Division of Reproductive Sciences, Columbia University College of Physicians and Surgeons, New York, NY 10032, USA; 2 Department of Physiology & Biophysics, University of Illinois Chicago, Chicago, IL 60612, USA; 3 Division of Reproductive Endocrinology and Infertility, Department of Obstetrics, Gynecology and Women's Health, Rutgers Biomedical and Health Sciences, Newark, NJ 07103, USA

**Keywords:** Trophoblasts, Notch, Placenta, *Cyp19-Cre*, *Tpbpa-Cre*

## Abstract

Proper development and function of the mammalian placenta requires interactions between embryo-derived trophoblasts and uterine endothelial cells to form mosaic vessels that facilitate blood flow to a developing conceptus. Notch signaling utilizes a cell–cell contact dependent mechanism to drive cell behaviors, such as differentiation and invasion. In mice, *Notch2* is needed for proper placentation and embryo survival. We used transgenic mice with a dominant-negative form of Mastermind-like1 and *Cyp19-Cre* and *Tpbpa-Cre* drivers to inhibit canonical Notch signaling in trophoblasts. Both *Cre* drivers resulted in robust placental expression of dominant-negative Mastermind-like1. All pregnancies progressed beyond mid-gestation and morphological analyses of placentas revealed no differences between mutants and controls. Our data suggest that mouse placentation occurs normally despite dominant negative inhibition of trophoblast canonical Notch signaling and that Notch2 signaling via the canonical pathway is not necessary for placentation.

## Abstract (precis)

**Summary:** Using transgenic mice with a dominant-negative form of Mastermind-like1 and *Cyp19-Cre* and *Tpbpa-Cre* drivers, we found that dominant negative inhibition of canonical Notch signaling in trophoblast cells does not disrupt placenta formation.

## INTRODUCTION

The development and function of the placenta relies heavily on embryo-derived trophoblasts (TBs), specialized cells that interact with maternal endothelial cells (ECs) to form mosaic vessels that facilitate proper blood flow to a developing conceptus (Cha et al., 2012; Cross et al., 1994; Hunkapiller and Fisher, 2008; Rai and Cross, 2014). In humans and mice, invasive TB subtypes mediate uterine vascular remodeling. Reduced TB-mediated uterine vascular invasion leads to poor placental vascular development and improper placentation, which are associated with miscarriages, intrauterine growth restriction and pre-eclampsia (Cha et al., 2012; Cross et al., 1994; Rossant and Cross, 2001). The signaling pathways active in the EC-TB crosstalk that underlies placentation have not been fully elucidated. However, Notch signaling, a regulator of angiogenesis and vascular remodeling, has been implicated in both human and mouse placenta formation (Chi et al., 2017; Gasperowicz and Otto, 2008; Gasperowicz et al., 2013; Haider et al., 2014, 2017; Hunkapiller et al., 2011; Kalkunte et al., 2017; Lee et al., 2018).

The Notch pathway regulates cell fate, cellular growth and invasion via direct cell-to-cell contact (Cuman et al., 2014; Kofler et al., 2011). Notch proteins (Notch1-4) are single-pass transmembrane receptors that are activated by membrane bound ligands of the Jagged and Delta-like ligand (Dll) families on adjacent cells (Shawber and Kitajewski, 2004). With canonical Notch signaling, binding of the ligand to Notch triggers cleavage and translocation of the Notch intracellular domain (NICD) to the nucleus, where it binds to the transcription factor recombination binding protein jκ (Rbpjκ). NICD binding and recruitment of co-activators, including Mastermind-like1 (MAML1), converts Rbpjκ from a transcriptional repressor to an activator, inducing downstream targets of the Hairy/Enhancer of Split (Hes) and Hairy/Enhancer of Split related with YRPW motif (Hey) families (Kofler et al., 2011; Kovall, 2007). In addition to Rbpjκ-mediated canonical Notch signaling, both the full-length Notch proteins and the NICD can signal via a non-canonical Rbpjk-independent pathway to regulate cell fate (Andersen et al., 2012; Shawber et al., 1996).

**Fig. 1.**
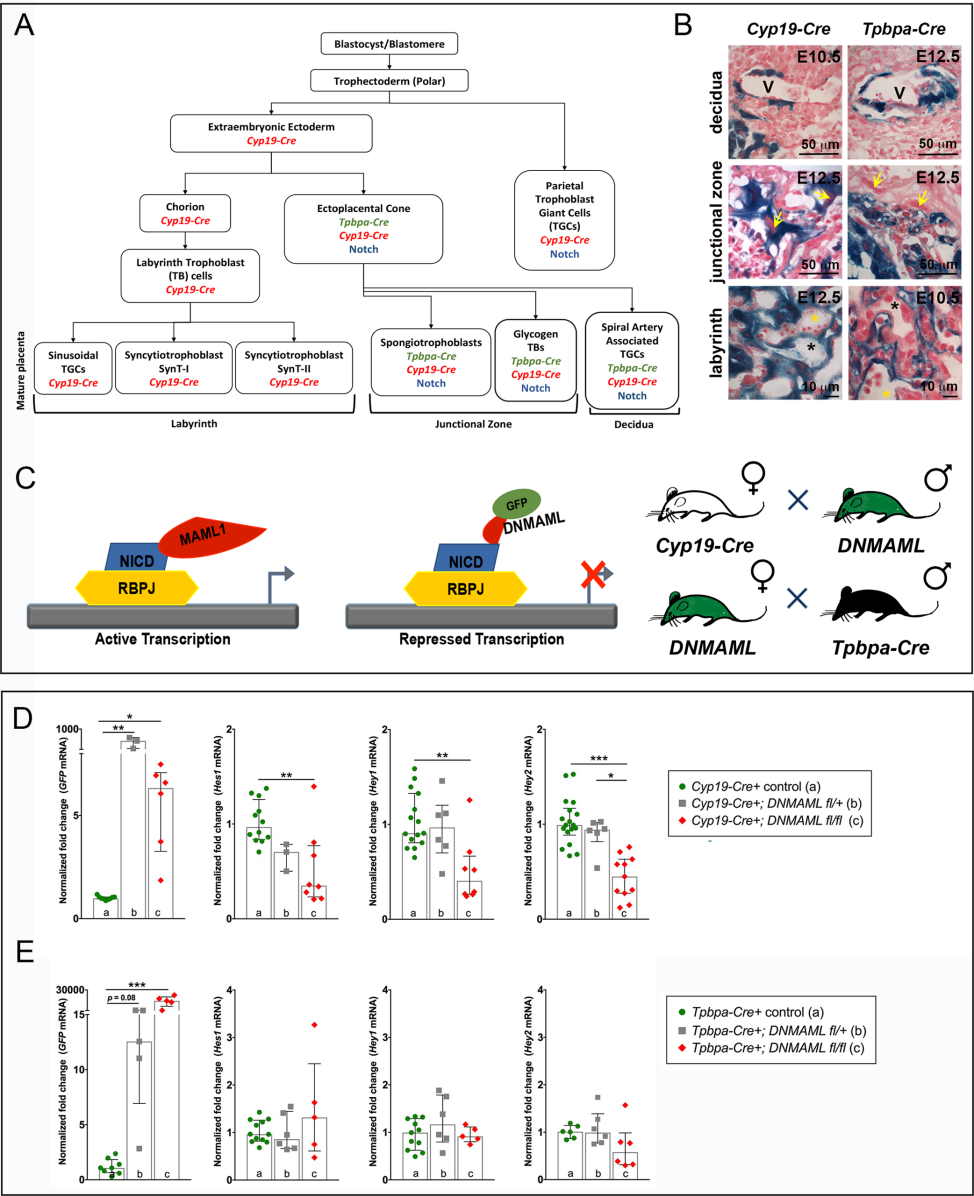
**Trophoblast-specific inhibition of canonical Notch signaling.** (A) Diagram of trophoblast (TB) differentiation and the overlap with TB-specific *Cyp19-Cre* and *Tpbpa-Cre* transgene expression and canonical Notch activity. (B) Representative images of X-gal and Nuclear Fast Red staining of *Cyp19-Cre;ROSA26 LacZ* placentas or *Tbpba-Cre;ROSA26 LacZ* placentas at E10.5 and E12.5. V marks maternal vessels with invading TBs. Yellow arrows mark parietal-TGCs. Yellow asterisks highlight fetal blood spaces. Black asterisks highlight maternal blood spaces. (C) Schematic of DNMAML inhibition of canonical Notch signaling. The Notch transactivation complex consists of the Notch intracellular domain (NICD) bound to Rbpjκ on the DNA, which recruits MAML1 to induce transcription of Notch target genes. DNMAML-GFP fusion protein binds the NICD, blocking assembly of the activation complex and inhibiting transcription of Notch target genes. Diagrams show sex of the mice used to produce *Cyp19-Cre* and *Tpbpa-Cre* mutant and control embryos. (D,E) Control and mutant E12.5 placentas were analyzed for expression of *GFP* (*DNMAML-GFP* fusion) and direct Notch targets, *Hes1*, *Hey1* and *Hey2* by qRT-PCR. Expression levels were normalized to 18 s rRNA. Data represent median and interquartile range. (D) Relative to *Cyp19-Cre+* placentas, *GFP* expression was significantly increased in *Cyp19-Cre+;DNMAML^fl/+^* (**P=0.002) and *Cyp19-Cre+;DNMAML^fl/fl^* (*P=0.02) placentas. *Hes1* (**P=0.008), *Hey1* (**P=0.006) and *Hey2* (***P<0.0001) expression was significantly decreased in *Cyp19-Cre+;DNMAML^fl/fl^* placentas compared to *Cyp19-Cre+* controls. *Hey2* expression was significantly decreased in *Cyp19-Cre+;DNMAML^fl/fl^* (*P*=0.05) compared to *Cyp19-Cre+;DNMAML^fl/+^* placentas. (E) *GFP* expression was significantly increased in *Tpbpa-Cre+;DNMAML^fl/fl^* placentas (***P=0.0006) relative to *Tpbpa-Cre+* controls. *Hes1*, *Hey1*, and *Hey2* expression did not differ between *Tpbpa-Cre+;DNMAML^fl/+^* and *Tpbpa-Cre+;DNMAML^fl/fl^* placentas relative to *Tpbpa-Cre+* controls.

In the mouse, Notch signaling is active in both ECs and TBs (Hunkapiller et al., 2011; Levin et al., 2017) and Notch function is critical for development of the placental vascular system and proper placenta formation (Chi et al., 2017; Gasperowicz and Otto, 2008; Haider et al., 2017; Hunkapiller et al., 2011). Notch expression and activity have been detected in TBs in the ectoplacental cone, TBs associated with maternal decidual vessels and TBs in the junctional zone (Hunkapiller et al., 2011; Levin et al., 2017) (Fig. 1A). Global deletion of *Notch1*, *Notch1/Notch4*, *Dll4*, or *Rbpj*κ resulted in embryonic lethality by mid-gestation via disruption in chorioallantoic branching and/or placental labyrinth network formation (Duarte et al., 2004; Gasperowicz and Otto, 2008; Krebs et al., 2000; Limbourg et al., 2005; Oka et al., 1995). Placentas lacking *Tle3*, a transcriptional co-repressor that interacts with downstream effectors of the Notch signaling pathway, have reduced junctional zone size and abnormal TB-lined maternal vasculature (Gasperowicz et al., 2013). *Notch2* null embryos died by embryonic day (E) 11.5 and whole embryo culture rescued lethality in mutant embryos, suggesting an extraembryonic cause (Hamada et al., 2007). Conditional deletion of *Notch2* with the TB-specific *Tpbpa-Cre* driver resulted in reduced size of maternal vessels and decreased placental perfusion, suggesting a requirement for *Notch2* in differentiation of invasive TB subtypes and TB-mediated remodeling of the maternal vasculature (Hunkapiller et al., 2011).

Herein, we used *Cyp19-Cre* and *Tpbpa-Cre*, two TB-specific Cre drivers with overlapping yet unique TB expression patterns, to investigate the requirement for TB-specific canonical Notch signaling in placentation and placental function. *Cyp19-Cre* induces recombination in many TB stem cell derivatives, including spongiotrophoblasts, labyrinthine TBs and many trophoblast giant cells (TGCs), including parietal-TGCs and spiral artery associated-TGCs (Moreau et al., 2014; Wenzel and Leone, 2007). *Tpbpa-Cre* expression includes invasive TB subtypes, glycogen trophoblast cells and spiral artery associated-TGCs, found in the mature placenta (Hu and Cross, 2010; Hunkapiller et al., 2011; Simmons et al., 2007). We hypothesized that placentation requires Notch in TBs and that inhibition of TB-specific canonical Notch signaling would severely impact placental development and pregnancy outcomes. In this study, we report the use of a dominant-negative form of MAML1 (DNMAML) to inhibit TB-specific canonical Notch signaling using both *Cyp19-Cre* and *Tpbpa-Cre* drivers. As the *Cyp19-Cre* transgene has broader expression, we predicted that the phenotype resulting from disrupted canonical Notch signaling could be more severe with the *Cyp19-Cre* driver than with the *Tpbpa-Cre* driver.

## RESULTS

### Trophoblast-specific expression of *Cyp19-Cre* and *Tpbpa-Cre* drivers

Prior to using *Cyp19-Cre* and *Tpbpa-Cre* drivers to investigate TB-specific Notch signaling (Fig. 1A), we confirmed the Cre activity in placental TB-subtypes by crossing each of the TB-specific *Cre* drivers with mice carrying the Cre reporter *ROSA26 LacZ* (Fig. 1B). X-gal staining showed *Cyp19-Cre* activity in TBs surrounding maternal vessels in the decidua, TBs in the junctional zone and in syncytiotrophoblasts in the labyrinth, consistent with previous reports (Wenzel and Leone, 2007). For *Tpbpa-Cre*, X-gal staining showed activity in TBs surrounding maternal vessels in the decidua and TBs in the junctional zone. Unexpectedly, we observed expression of the *Tpbpa-Cre* transgene associated with the fetal vasculature, suggesting a broader expression pattern of this transgene than initially reported.

### Trophoblast-specific expression of *DNMAML* does not prevent progression of pregnancy

To evaluate the requirement for TB-specific canonical Notch signaling in pregnancy, we crossed *Cyp19-Cre* and *Tpbpa-Cre* mice with a transgenic mouse line expressing dominant-negative MAML1/GFP fusion protein (*DNMAML^fl/fl^*). DNMAML forms an inactive complex with NICD/Rbpjκ, inhibiting Rbpjκ-dependent transcriptional activation downstream of all Notch proteins (Fig. 1C). This mating strategy will block TB-specific canonical Notch signaling during placentation. To confirm *DNMAML* transgene expression, we harvested placentas at E12.5, isolated RNA from whole placentas and determined *GFP* transcript levels by qRT-PCR. We observed a significant increase in *GFP* expression in *Cyp19-Cre*+;*DNMAML^fl/+^* and *Cyp19-Cre*+;*DNMAML^fl/fl^* placentas compared to the *Cyp19-Cre*+ controls and in *Tpbpa-Cre*+;*DNMAML^fl/fl^* placentas compared to *Tpbpa-Cre*+ controls (Fig. 1D,E). To verify the ability of DNMAML to inhibit Notch signaling in the placenta, transcript levels of *Hes1*, *Hey1* and *Hey2*, direct targets of canonical Notch signaling, were assessed (Fig. 1D,E). *Hes1*, *Hey1*, and *Hey2* were significantly decreased in *Cyp19-Cre*+;*DNMAML^fl/fl^* placentas compared to *Cyp19-Cre*+ controls. The lack of reduction in Notch effector expression in *Cyp19-Cre*+;*DNMAML^fl/+^* whole placentas may be due to mosaic recombination of only one DNMAML allele, and thus lower expression levels of the mutant protein (see Fig. 1D). In contrast, there were no significant changes in expression of the Notch targets in either *Tpbpa-Cre*+;*DNMAML^fl/+^* or *Tpbpa-Cre*+;*DNMAML^fl/fl^* mutant placentas compared to *Tpbpa-Cre*+ controls.

To determine if TB-specific expression of DNMAML affected progression of pregnancy, we collected and weighed embryos and placentas at various developmental stages from E10.5 to E16.5. For all crosses, mid-gestation median litters sizes (Table S1) were similar to those for C57BL/6J pregnancies in our colony (Levin et al., 2017). We found that resorption rates for E12.5 litters with *Cyp19-Cre* or *Tpbpa-Cre* mediated expression of DNMAML did not exceed 11% (data not shown). TB-specific inhibition of *Notch2* with the *Tpbpa-Cre* driver resulted in 29–63% embryonic loss by E12.5 (Hunkapiller et al., 2011). Thus, we observed less embryonic death at E12.5 than was observed with the *Notch2* TB-deletion. These data suggest that TB-specific expression of DNMAML did not negatively influence the overall progression of pregnancy to mid-gestation.

### Dominant negative inhibition of canonical Notch signaling with the *Cyp19-Cre* driver does not alter placentation

**Fig. 2.**
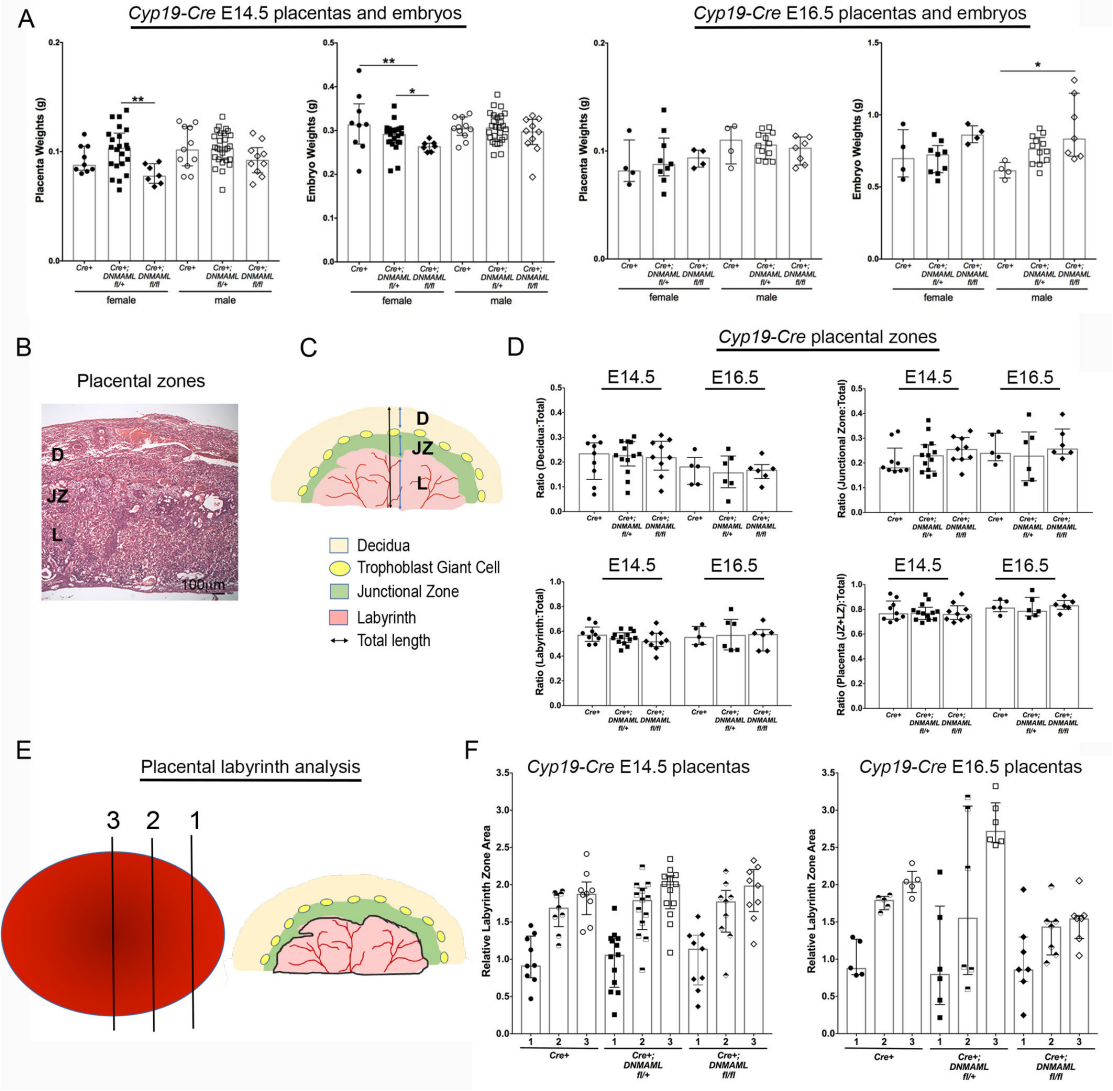
**Assessment of pregnancy and placental morphology with *Cyp19-Cre* mediated canonical Notch inhibition.** (A) Weights of *Cyp19-Cre+*, *Cyp19-Cre*+;*DNMAML^fl/+^*, *and Cyp19-Cre*+;*DNMAML^fl/fl^* placentas at E14.5 and E16.5. (B) Representation of the H&E stained placental sections used for analyses of placental zones: D, decidua; JZ, junctional zone; L, labyrinth. Scale bar: 100 µm. (C) Diagram of a mature placenta. The total thickness of the placenta and the depth of each placental zone (blue color lines) were measured and expressed as the ratio of the depth of each zone to the total placental depth. (D) At E14.5 and E16.5, the fractional depth occupied by each placental zone was similar in *Cyp19-Cre+;DNMAML^fl/+^* and *Cyp19-Cre+;DNMAML^fl/fl^* mutant placentas relative to *Cyp19-Cre+* controls. (E) Diagram illustrating half of the placental disk divided into three equal portions. Data are presented as a ratio of labyrinth areas 2 or 3 relative to labyrinth area 1. (F) At E14.5 and E16.5, normalized labyrinth areas 2 and 3 were similar in *Cyp19-Cre+;DNMAML^fl/+^* and *Cyp19-Cre+;DNMAML^fl/fl^* mutant placentas relative to controls. Data are presented as median and interquartile range. *P<0.05, **P<0.01.

To investigate the effect of *Cyp19-Cre* mediated inhibition of canonical Notch signaling on embryonic and placental development, we assessed *Cyp19-Cre* pregnancies at mid- to late-gestational stages, E14.5 and E16.5 for sex-specific differences in embryo and placenta weights (Fig. 2A). At E14.5, inhibition of canonical Notch signaling in TBs expressing *Cyp19-Cre* had no effect on male embryo or placenta weights. At E16.5, male placenta weights were also similar, but *Cyp19-Cre*+;*DNMAML^fl/fl^* male embryos were larger than littermate controls. For females, we found that at E14.5, *Cyp19-Cre*+;*DNMAML^fl/fl^* placentas and embryos were significantly smaller than *Cyp19-Cre*+;*DNMAML^fl/+^* placentas and embryos, and *Cyp19-Cre*+;*DNMAML^fl/fl^* embryos were significantly smaller than their littermate controls. However, by E16.5, weights of female embryos and placentas from mutants and controls were similar. These results suggest that TB-specific inhibition of Notch signaling by *Cyp19-Cre* may be having an effect on female embryo weights at E14.5, but the embryo weights recover later in pregnancy.

To determine the effect of *Cyp19-Cre* mediated inhibition of canonical Notch signaling on placental morphology, we assessed formation of placental zones and labyrinth size at mid- to late-gestation. The fully developed mouse placenta contains three major zones, the maternal decidua (D), the junctional zone (JZ), and the labyrinth (L) (Fig. 2B,C). The labyrinth is the placental region responsible for nutrient and gas supply to the fetus during pregnancy, a key determinant of fetal growth and development (Rossant and Cross, 2001). Thus, we performed additional morphometric analyses of the labyrinth area to determine if there were any subtle structural changes. Sections from the central region of the placenta were stained with Hematoxylin and Eosin (H&E) (Fig. 2B). To assess formation of placental zones, the depth of each zone was measured as illustrated in Fig. 2C and the ratio relative to the total placental depth (D+JZ+L) was determined. To assess the labyrinth, the hemi-placenta was divided into three even portions (see Fig. 2E). A representative section from each portion was H&E stained and the relative labyrinth area of each section was determined (Cui et al., 2013).

To determine if the smaller placental weights we observed for *Cyp19-Cre*+;*DNMAML^fl/fl^* female placentas were associated with altered placental morphology, we compared placental zone size and labyrinth areas for female embryos at E14.5. We found that sizes of all placental zones and relative labyrinth zone areas were similar for *Cyp19-Cre*+;*DNMAML^fl/fl^* and *Cyp19-Cre*+;*DNMAML^fl/+^* mutants and *Cyp19-Cre*+ controls (Fig. S1A,B). We concluded that although placental mass was decreased for *Cyp19-Cre*+;*DNMAML^fl/fl^* female embryos at E14.5, similar placental morphology combined with normalization of placental weights by E16.5 did not support sexually dimorphic placental development. Thus, we combined data for male and female embryos for the subsequent analyses of placental morphology. We did not detect any differences in the sizes of *Cyp19-Cre*+;*DNMAML^fl/+^* and *Cyp19-Cre*+;*DNMAML^fl/fl^* placental zones at E14.5 or E16.5 as compared to *Cyp19-Cre*+ littermate controls (Fig. 2D). Analyses of the relative labyrinth zone areas showed no differences between mutants and littermate controls for *Cyp19-Cre* placentas at E14.5 and E16.5 (Fig. 2F). Taken together, these data suggest that inhibition of canonical Notch signaling in TBs with the *Cyp19-Cre* driver did not affect development of placental zones and did not result in an expanded or smaller placental labyrinth. These data also suggest that canonical Notch signaling is not necessary for the function of TB cells in the labyrinth.

### *Tpbpa-Cre* mediated expression of *DNMAML* does not impair placentation and embryonic growth

To determine if TB-specific expression of DNMAML affected placentation, we assessed *Tpbpa-Cre+* pregnancies for sex-specific differences in placenta or embryo weights and differences in placental morphology. At E12.5, placenta and embryo weights were similar for *Tpbpa-Cre+*;*DNMAML^fl/+^* and *Tpbpa-Cre*+;*DNMAML^fl/fl^* mutant and littermate control females and males (Fig. S2A). We further assessed gross placental morphology with H&E staining. At E12.5, we did not detect any differences in the placental zones (Fig. S2B) or relative labyrinth areas (Fig. S2C) of *Tpbpa-Cre+* mutant placentas as compared to littermate controls. The absence of a phenotype was consistent with the qRT-PCR analyses of E12.5 *Tpbpa-Cre;DNMAML^fl/fl^* whole placentas in which expression of Notch effectors was not affected, although DNMAML expression was determined (Fig. 1E). Thus, *Tpbpa-Cre;DNMAML^fl/fl^* did not inhibit canonical Notch signaling in trophoblasts resulting in an absence of a placental or pregnancy phenotype.

### Minimal overlap between Notch activity and TB-specific Cre transgene expression in the junctional zone

**Fig. 3.**
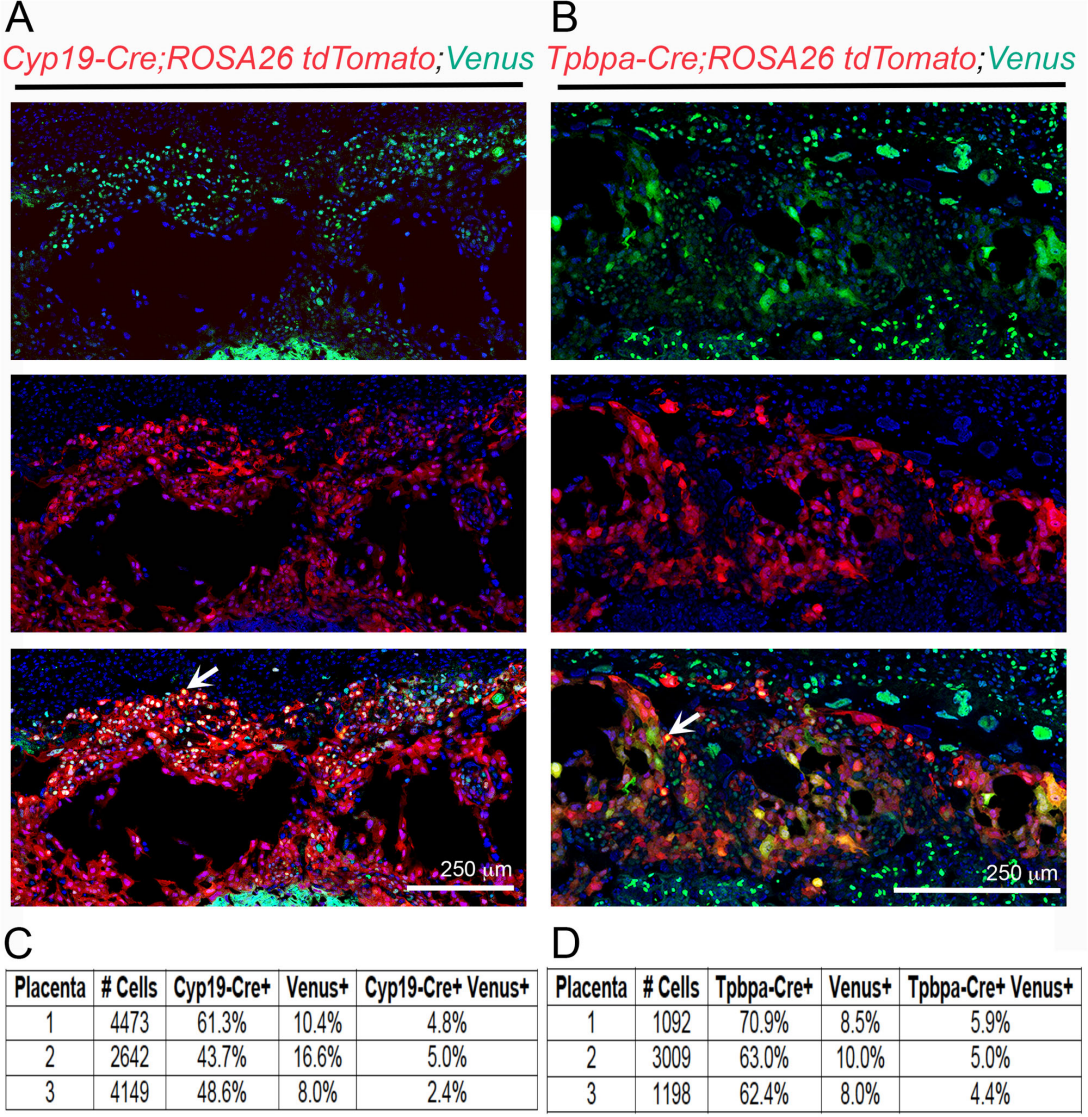
**Trophoblast-specific *Cre* drivers have low incidence of overlap with canonical Notch signaling in the junctional zone.** Representative images of E12.5 placental sections with Venus-YFP (green) indicating canonical Notch activity, tdTomato (red) indicating *Cyp19-Cre* expression (A) or *Tpbpa-Cre* expression (B), and co-expression (yellow, arrows) of *Cyp19-Cre; ROSA26 tdTomato* with Notch-*Venus* (A) or *Tpbpa-Cre; ROSA26 tdTomato* with Notch-*Venus* (B) are shown. Nuclei were stained with DAPI (blue). Scale bars: 250 µm. (C,D) Percentages of single positive, *Cyp19-Cre+* or *Tpbpa-Cre+* or *Venus*-YFP+ and percentages of double positive, *Cyp19-Cre+ Venus*-YFP+ and *Tpbpa-Cre*+ *Venus*-YFP+ cells were determined for three placentas for each *Cre* driver.

Our studies suggest that canonical Notch signaling is not required for TB invasion and placental development. To evaluate the overlap between junctional zone TBs with active Notch signaling and *Cyp19-Cre* or *Tpbpa-Cre* transgene expression, *Cyp19-Cre;ROSA26 tdTomato* or *Tpbpa-Cre;ROSA26 tdTomato* reporter mice were mated to the Notch-*Venus* reporter line that expresses YFP in the presence of canonical Notch signaling and placental sections were assessed (Fig. 3A,B). At E12.5, *Cyp19-Cre+* and Notch signaling overlapped in 4.8% (IQR 2.4, 5.0) TBs in the junctional zone (Fig. 3A,C) and co-expression of *Tpbpa-Cre*+ and Notch signaling was observed in 5.0% (IQR 4.4, 5.9) TBs in the junctional zone (Fig. 3B,D). The low incidence of overlap presents limited opportunities for DNMAML to inhibit canonical Notch signaling activity in the junctional zone and may explain the absence of impaired placentation and poor pregnancy outcomes in our models.

## DISCUSSION

During placentation, multiple TB subtypes have been shown to undergo canonical Notch signaling (Hunkapiller et al., 2011; Levin et al., 2017). We previously showed canonical Notch signaling and overlapping expression of Notch2 and Notch4 in junctional zone TBs (Levin et al., 2017), suggesting that in TBs Notch2 and Notch4 signal via canonical Notch signaling to mediate formation of the placental vasculature. Consistent with this idea, deletion of *Notch2* in invasive TBs reduced the size of maternal blood canals and decreased placental perfusion, leading to increased embryonic loss by mid-gestation (Hunkapiller et al., 2011). In contrast, we found here that expression of *DNMAML*, an antagonist of canonical Notch signaling, under either *Cyp19* or *Tpbpa* promoters did not significantly affect placenta formation or pregnancy progression.

In the *Cyp19-Cre* model, expression of *DNMAML* correlated with decreased expression of Notch effectors at E12.5, reflecting loss of TB-specific canonical Notch signaling activity. At E14.5, we found that *Cyp19-Cre*+ mutant female placentas and embryos were smaller than their littermate controls. However, both placental and embryo weights normalized by late-gestation. As we found no differences in placental morphology, it is possible that the pregnancies could overcome these defects. In contrast to the *Cyp19cre;DNMAML* females, no differences were observed in the placentas and embryo weights of male mutant and control embryos at all stages of gestation. This difference between male and female embryos may be due to sexual dimorphism in placentation. In support of this, it has been proposed that altered differentiation of invasive TB lineages could perturb uterine vascular remodeling in a sex-specific way, resulting in altered junctional zone and labyrinth structure and function (Kalisch-Smith et al., 2017). However, sex-specific differences in placental structure often manifest in late-gestation (Gabory et al., 2013; Kalisch-Smith et al., 2017), and thus our findings may arise from a different cause.

In the *Tpbpa-Cre* model, we also observed *DNMAML* expression in the placentas of mutants. However, expression of *DNMAML* in *Tpbpa-Cre*+ TBs did not reduce the expression of Notch targets in whole placentas. The lack of effect of *Tpbpa-Cre* mediated *DNMAML* expression on placenta and embryo weights and placenta morphology is consistent with the lack of effect on Notch signaling activity. We confirmed that our *Tpbpa-Cre* driver was expressed in junctional zone TB subtypes and spiral artery associated-TGCs, as previously described (Hu and Cross, 2010; Hunkapiller et al., 2011; Simmons et al., 2007). Further, we found similar overlap between Notch signaling activity and recombination in both the *Cyp19-Cre* and *Tpbpa-Cre* drivers. Thus, it still remains unclear why a loss of Notch effectors was not observed in the *Tpbpa-Cre;DNMAML* studies.

The cause for the difference between our findings in the *Cyp19-Cre;DNMAML* model and the reported *Tpbpa-Cre;Notch2^fl/fl^* results remains to be determined. One possibility is that Notch2 signaling does not require activation of the canonical pathway in placentation (Gasperowicz and Otto, 2008; Hunkapiller et al., 2011). However, our findings may be the result of differences in the animal facilities with different predominant microbiomes, differences in mouse genetic background or differences in the type of transgenic models used.

Our model of TB-specific deletion of canonical Notch signaling with *DNMAML* raises the possibility of a more complex underlying mechanism in which non-canonical Notch signaling is sufficient to allow for TB differentiation, whereas canonical Notch signaling is not necessary for placentation. Studies on the role of non-canonical Notch signaling in placental development are needed to further our understanding of the role of Notch in placentation.

## MATERIALS AND METHODS

### Animals

The Columbia University Institutional Animal Care and Use Committee (AC-AAAQ2407) approved the studies. *Cyp19-Cre* mice [Tg(Cyp19a1-cre)5912Gle] were a gift from Dr Gustavo Leone (The Ohio State University, USA) and were maintained on a FVB genetic background (Wenzel and Leone, 2007). *Tpbpa-Cre* mice [STOCK-Tg(Tpbpa-cre, -EGFP)5Jcc] (Simmons et al., 2007) were purchased from the Canadian Mutant Mouse Repository after recovery of the line from cryopreserved sperm. *Tpbpa-Cre* mice were backcrossed seven generations unto a C57BL/6J background and maintained on this background for all experiments. The *Cyp19-Cre* transgene shows the most consistent placental expression when inherited through the maternal line (Wenzel and Leone, 2007). The *Tpbpa-Cre* transgene has nonspecific promoter activity, with ovarian expression, when inherited through the maternal line (Hunkapiller et al., 2011). To obtain optimal, cell-type specific Cre activity, we used *Cyp19-Cre+* females and *Tpbpa-Cre+* males for all experiments (Fig. 1C; Table S1).

*ROSA26-DNMAML1-GFP* mice, hereafter referred to as *DNMAML*, were a gift from Dr Warren Pear (The University of Pennsylvania, USA) (Tu et al., 2005). *B6.Cg-Gt(ROSA)26Sor^tm14(CAG-tdTomato)Hze^/J* reporter mice (*ROSA26 tdTomato*) *and Rosa-lox-stop-lox-LacZ* reporter mice (*ROSA26 LacZ*) were obtained from Jackson Laboratories and maintained on a C57BL/6 background. The Notch reporter line *CBF:H2B-Venus* mice, hereafter referred to as Notch-Venus, were also obtained from Jackson Laboratories and maintained on a C57BL/6 background (Nowotschin et al., 2013).

Noon on the day a mating plug was observed was designated as E0.5. Placentas and embryos were analyzed at E10.5, E12.5, E14.5 and E16.5. The wet weight of each embryo and its corresponding placenta were recorded. Half of each placenta was fixed in 4% paraformaldehyde (PFA) and half was fixed in Bouins Solution (Sigma-Aldrich) or snap frozen on dry ice and stored at −80°C for RNA extraction. Determination of embryo sex with primers specific for the sex-determining gene on the Y chromosome (Sry) allowed for comparisons of embryo weights and placental phenotypes for female and male sex. Genotyping and sex determination were performed using the PCR primers listed in Table S2.

### Quantitative reverse transcription-PCR

Total RNA was extracted from placentas using TRIzol® (Invitrogen) and reverse transcribed using qScript cDNA Supermix (Quanta Biosciences). Gene expression was determined by quantitative (q) RT-PCR using the QuantiNova SYBR Green PCR Kit (Qiagen, Frederick, MD, USA) and primer sequences listed in Table S3. Each qRT-PCR was performed in triplicate with 25 ng cDNA per reaction. The relative expression levels of the target genes were quantified using the 2^−ΔΔCT^ method (Lacko et al., 2014) and expressed as fold change normalized to 18 s rRNA (Sones et al., 2018).

### Evaluation of *Cyp19-Cre* and *Tpbpa-Cre* activity

To confirm TB-specific Cre activity, *Cyp19-Cre+* females were mated with *ROSA26 LacZ* reporter males and *Tpbpa-Cre+* males were mated with *ROSA26 LacZ* reporter females. Placentas were harvested, fixed in 4% PFA for 1 h, infiltrated with 30% sucrose, embedded in Tissue-Tek OCT Compound (Sakura Fine Technical, Torrance, CA, USA), and cryosectioned in the longitudinal plane at 10 µm. Sections were stained with X-gal and counterstained with Nuclear Fast Red (Sigma-Aldrich) (Soriano, 1999). X-gal staining demonstrated β-galactosidase activity, which represents Cre activity. Sections were examined with a Nikon MICROPHOT-FXA microscope and images were captured using NIS-Elements D3.10 software.

### Evaluation of *Cyp19-Cre* or *Tpbpa-Cre* transgene expression and canonical Notch signaling

For assessment of placental Notch signaling activity with respect to Cre expression, *Cyp19-Cre;ROSA26 tdTomato* and *Tpbpa-Cre;ROSA26 tdTomato* reporter mice were mated with Notch-Venus mice. Venus-yellow fluorescent protein (YFP) expression provides a quantitative, single-cell resolution read out of canonical Notch signaling (Levin et al., 2017; Nowotschin et al., 2013). Placentas were harvested at E12.5, fixed in 4% PFA, and processed as described above. Vectashield with 4′, 6-diamidino-2-phenylindole (DAPI) (Vector Labs) was used for mounting and visualizing nuclei (blue). For assessment of Notch signaling activity with respect to *Cre* expression in the junctional zone, the entire junctional zone was imaged. Four to six areas, in which *Cyp19-Cre;ROSA26 tdTomato* or *Tpbpa-Cre;ROSA26 tdTomato* and Notch-Venus signal expression were most identifiable, were selected for each junctional zone. At least 1000 cells were counted. We identified junctional zone cells with *Cre* expression or canonical Notch signaling activity and cells with both *Cre* expression and canonical Notch signaling activity. The percentages of single positive, *Cyp19-Cre+* (red) or *Tpbpa-Cre+* (red) or *Venus*-YFP+ (green), and the percentages of double positive, *Cyp19-Cre+ Venus*-YFP+ (yellow) and *Tpbpa-Cre*+ *Venus*-YFP+ (yellow), cells were calculated. Three different placentas from each *Cre* line were evaluated. Images were captured using a Nikon A1 scanning confocal microscope on an Eclipse Ti microscope stand (Nikon Instruments, Melville, NY, USA) and analyzed with ImageJ software (NIH).

### Placental histological analysis

To evaluate placental morphology, tissues fixed in Bouins solution were embedded in paraffin, sectioned at 10 µm, and stained with H&E. Sectioned placentas were assessed from the central region of the placenta, determined by identifying the thickest section and presence of umbilical cord attachment. Two analyses of placental morphology were performed. (1) To determine the relative cross-sectional area of each placental zone, decidua (D), junctional zone (JZ) and labyrinth (L), the midpoint of the thickest placental section was determined and the depth of each zone was measured (Fig. 2C) (Dokras et al., 2006). The depth of each zone was expressed as the ratio of that zone to the total placental thickness (D+JZ+L). (2) To determine the relative area of the labyrinth, placentas were assessed as previously described (Cui et al., 2013). The thickest placental section was identified and the hemi-placenta was divided into thirds (Fig. 2E). The labyrinth was outlined on a representative section from each third. To control for variability in sectioning and the subsequent lack of uniformity in identification of the first portion of each placenta, the mean of the first labyrinth zone area was calculated for each genotype and used as a normalizer for that genotype. To allow for comparison of change in median labyrinth zone areas 2 and 3 across genotypes, each individual labyrinth zone 2 and 3 was divided by the normalizer for that genotype. A minimum of five placentas were analyzed for *Cyp19-Cre* control and mutant genotypes and a minimum of three placentas were analyzed for *Tpbpa-Cre* control and mutant genotypes. Images were captured and measurements were made with NIS-Elements D3.10 software.

### Statistics

Non-parametric statistical analyses were performed with Prism v6.0f (GraphPad Software, La Jolla, CA, USA). We analyzed these data to identify outliers. Medians were compared with Kruskal–Wallis and Dunn's multiple comparisons tests. Data are expressed as median with interquartile range (IQR). Statistical significance was defined as *P*<0.05.

## Supplementary Material

Supplementary information
