# Supplementary material for: Dominant-negative inhibition of canonical Notch signaling in trophoblast cells does not disrupt placenta formation
Source: Biol Open. 2019 Apr 10;8(4):bio037721. doi: 10.1242/bio.037721 (PMC6504009; doi:10.1242/bio.037721)
Supplement: Supplementary information [file biolopen-8-037721-s1.pdf]

Biology Open (2019): doi:10.1242/bio.037721: Supplementary information

Table S1. Mouse crosses used to inhibit canonical Notch signaling in placental trophoblasts

MALE	FEMALE	No. embryos at mid-gestation*	CONTROL GENOTYPES	MUTANT GENOTYPES
C57BL/6J	*Cyp19-Cre+;DNMAML$^{fl/+}$*	10 (9.5, 10.5)	*Cyp19-Cre+*<br>*DNMAML$^{fl/+}$*	*Cyp19-Cre+;DNMAML$^{fl/+}$*
DNMAML$^{fl/fl}$	*Cyp19-Cre+;DNMAML$^{fl/+}$*	9.5 (7.5, 11.8)	*Cyp19-Cre+*	*Cyp19-Cre+;DNMAML$^{fl/+}$*<br>*Cyp19-Cre+;DNMAML$^{fl/fl}$*
Tpbpa-Cre+;DNMAML$^{fl/+}$	*C57BL/6J*	8.5 (8, 9.8)	*Tpbpa-Cre+*<br>*DNMAML$^{fl/+}$*	*Tpbpa-Cre+;DNMAML$^{fl/+}$*
Tpbpa-Cre+;DNMAML$^{fl/+}$	*DNMAML$^{fl/+}$*	7 (3.5, 9)	*Tpbpa-Cre+*	*Tpbpa-Cre+;DNMAML$^{fl/+}$*<br>*Tpbpa-Cre+;DNMAML$^{fl/fl}$*

*Median (25th percentile, 75th percentile) number of embryos recovered from 4-8 litters per cross at E12.5 – E14.5 was similar to median litters sizes for C57BL/6 pregnancies, 9 (8, 11) embryos.

Biology Open • Supplementary information

Table S2. PCR Primers for genotyping

Target	Forward 5' -> 3'	Reverse 5' -> 3'	Product size	Source
Generic Cre	GCG GCA TGG TGC AAG TTG AAT	CGT TCA CCG GCA TCA ACG TTT	300 bp	MGI: 5287872
Cyp19-Cre	GAC CTT GCT GAG ATT AGA TC	GAC GAT GAA GCA TGT TTA GCT GGC C	545 bp	Wenzel, P.L. et al., 2007 PMID: 17299749
ROSA26;LacZ, Rosa;DNMAML	WT: AAA GTC GCT CTG AGT TGT TAT	TAA GCC TGC CCA GAA GAC TC	235 bp	Douglas, N.C. et al., 2013 PMID: 24089201
	MUT: GCG AAG AGT TTG TCC TCA ACC		309 bp	
CBF:H2B-Venus	AAG TTC ATC TGC ACC ACC G	TGC TCA GGT AGT GGT TGT CG	475 bp	Shawber, C.J. et al. 2015 PMID: 26629328
ROSA26 tdTomato	WT: AAG GGA GCT GCA GTG GAG TA	WT: CCG AAA ATC TGT GGG AAG TC	300 bp	Madisen, L. et al., 2009 PMID: 20023653 JAX Stock Number:007914

MUT: mutant; WT: wild type

Table S3. Primers for quantitative RT-PCR

Target	Forward 5' -> 3'	Reverse 5' -> 3'	Source
18s	CCG GGC TTC TAT TTT GTT GGT	TAG CGG CGC AAT ACG AAT G	Sones, J.L. et al., 2017<br>PMID: 29279353
Hes1	CCA GCC AGT GTC AAC ACG A	AAT GCC GGG AGC TAT CTT TCT	Thomas, M.M. et al., 2014<br>PMID: 25416148
Hey1	GCG CGG ACG AGA ATG GAA A	TCA GGT GAT CCA CAG TCA TCT G	
Hey2	AAG CGC CCT TGT GAG GAA AC	GGT AGT TGT CGG TGA ATT GGA C	
GFP	AAG TTC ATC TGC ACC ACC G	TCC TTG AAG AAG ATG GTG CG	Chan, K.M. et al., 2011<br>PMID: 21940502

Biology Open • Supplementary information

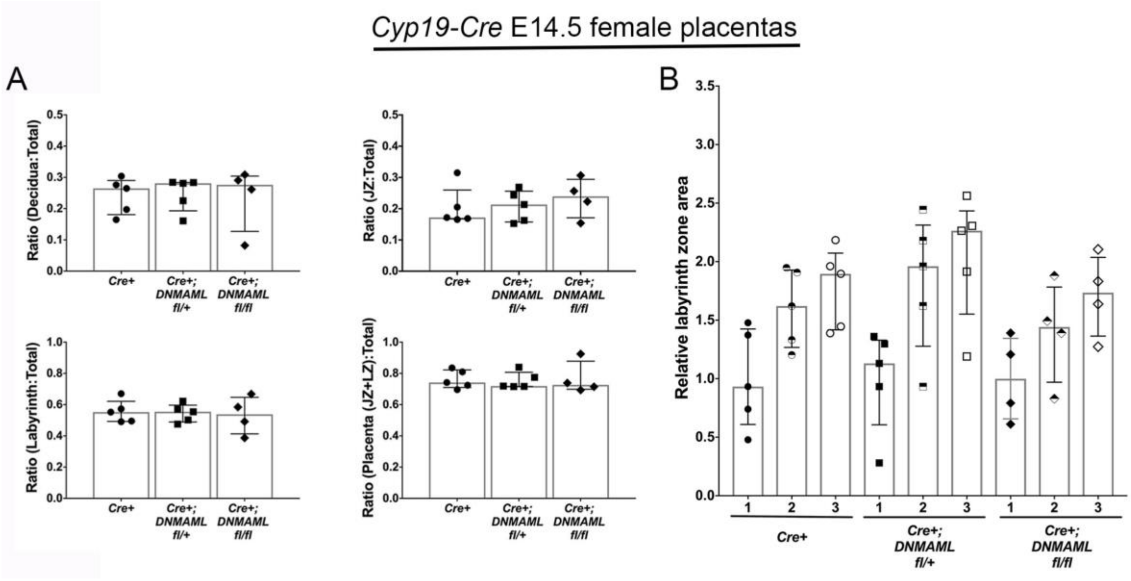

Figure S1. Trophoblast-specific deletion of canonical Notch signaling with the *Cyp19-Cre* transgene does not affect gross morphology of female placentas. *Cyp19-Cre* mice were crossed with *DNMAML* mice. Placentas from female embryos were compared. (A) Conditional inhibition of canonical Notch signaling with the *Cyp19-Cre* driver did not affect the size of any placental zone at E14.5. (B) Quantitative analyses of the relative labyrinth zone areas showed no difference for *Cyp19-Cre;DNMAML*$^{fl/+}$ or *Cyp19-Cre;DNMAML*$^{fl/fl}$ mutants as compared to *Cyp19-Cre*+ controls compared at E14.5. Data are presented as median and interquartile range.

Biology Open • Supplementary information

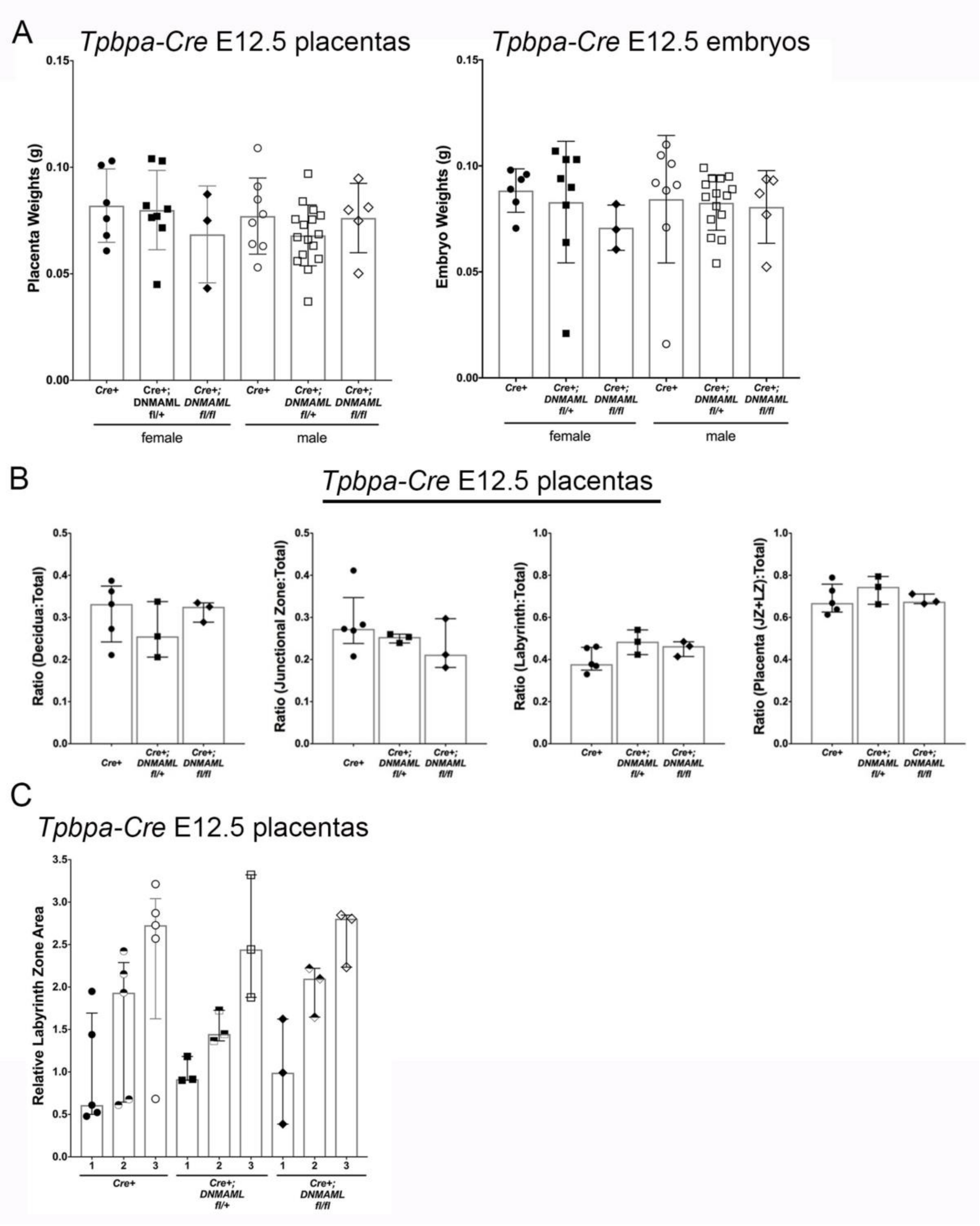

Figure S2. Assessment of pregnancy and placental morphology with *Tpbpa-Cre* mediated canonical Notch inhibition. *Tpbpa-Cre* mice were crossed with *DNMAML* mice. Placentas and embryos were collected and weighed. (A) Placenta and embryo weights for *Tpbpa-Cre;DNMAML*$^{fl/+}$ or

Biology Open • Supplementary information

Tpbpa-Cre;DNMAML$^{fl/fl}$ mutants were similar to *Tpbpa-Cre*+ controls *at* E12.5. (B) Conditional inhibition of canonical Notch signaling with the *Tpbpa-Cre* driver did not affect the size of any placental zone at E12.5. (C) Quantitative analyses of the relative labyrinth zone areas showed no difference for *Tpbpa-Cre;DNMAML*$^{fl/+}$ or *Tpbpa-Cre;DNMAML*$^{fl/fl}$ mutants as compared to *Tpbpa-Cre*+ controls compared at E12.5. Data are presented as median and interquartile range.
